# Unlocking the potential of SnS_2_: Transition metal catalyzed utilization of reversible conversion and alloying reactions

**DOI:** 10.1038/srep41015

**Published:** 2017-01-19

**Authors:** Zhi Xiang Huang, Ye Wang, Bo Liu, Dezhi Kong, Jun Zhang, Tupei Chen, Hui Ying Yang

**Affiliations:** 1 Pillar of Engineering Product Development, Singapore University of Technology and Design, 8 Somapah Road, Singapore 487372, Singapore; 2 Airbus Group Innovations Singapore, 110 Seletar Aerospace View 797562, Singapore; 3 School of Electrical and Electronic Engineering, Nanyang Technological University, Singapore 639798, Singapore

## Abstract

The alloying-dealloying reactions of SnS_2_ proceeds with the initial conversion reaction of SnS_2_ with lithium that produces Li_2_S. Unfortunately, due to the electrochemical inactivity of Li_2_S, the conversion reaction of SnS_2_ is irreversible, which significantly limit its potential applications in lithium-ion batteries. Herein, a systematic understanding of transition metal molybdenum (Mo) as a catalyst in SnS_2_ anode is presented. It is found that Mo catalyst is able to efficiently promote the reversible conversion of Sn to SnS_2_. This leads to the utilization of both conversion and alloying reactions in SnS_2_ that greatly increases lithium storage capability of SnS_2_. Mo catalyst is introduced in the form of MoS_2_ grown directly onto self-assembled vertical SnS_2_ nanosheets that anchors on three-dimensional graphene (3DG) creating a hierarchal nanostructured named as SnS_2_/MoS_2_/3DG. The catalytic effect results in a significantly enhanced electrochemical properties of SnS_2_/MoS_2_/3DG; a high initial Coulombic efficiency (81.5%) and high discharge capacities of 960.5 and 495.6 mA h g^−1^ at current densities of 50 and 1000 mA g^−1^, respectively. Post cycling investigations using *ex situ* TEM and XPS analysis verifies the successful conversion reaction of SnS_2_ mediated by Mo. The successful integration of catalyst on alloying type metal sulfide anode creates a new avenue towards high energy density lithium anodes.

The relentless growth of modern technology is constantly feeding society’s increasing need for energy usage and consumption. However, the development of energy storage devices has not been able to keep pace with the rapid progress of portable electronics, large-scale usage such as Electric Vehicles (EVs), plug-in hybrid Electric Vehicles (PHEV) and even grid-scale storage^1^,^2^,^3^. Over the course of developing an effective anode material, tin-based anode materials has been recognized as one of the most promising materials to replace the current generation of commercial graphite anode^4^,^5^,^6^. This is due to its salient features such as high theoretical capacity, high energy density owing to low voltage discharge profile and low cost due to its high abundance in nature^6^,^7^. Amongst the different tin-based anode materials, SnS_2_ offers a two-dimensional layered type structure where the CdI_2_-type layers are loosely bounded by weak van der Waals forces and hence easily susceptible to the intercalation of lithium-ions (Li_x_SnS_2_)^8^,^9^. Further interaction with lithium-ions in a discharge process results in the conversion process of the lithiated SnS_2_ to form metallic Sn and Li_2_S^8^,^10^. In subsequent charge-discharge cycles, the liberated tin alloys reversibly with lithium forming Li_4.4_Sn. The above reactions can be described using the following equations:


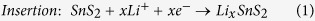



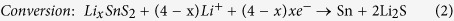


Simplifying equations (1) & (2),


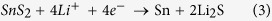


In subsequent charge-discharge cycles,


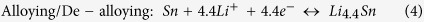


The reversible reaction of Sn in SnS_2_ with 4.4 mols of lithium (equation (4)) translates into a high theoretical capacity of 645 mAh g^−1^ (compared to graphite: 372 mAh g^−1^). Despite this, it can be observed that 4 mols of lithium is spent in the irreversible formation of Li_2_S during the conversion reaction of lithiated SnS_2_ (equation 2). Hence, the theoretical capacity of SnS_2_ can potentially be as high as 1231 mAh g^−1^ (8.4 mol Li^+^ per mol SnS_2_) compared to 645 mAh g^−1^ (4.4 mol Li^+^ per mol SnS_2_) if the irreversible reaction in equation 2 is made reversible.

To date, there are several reports of SnS_2_ with beyond theoretical capacities of 645 mAh g^−1^ 11,12,13,14,15,16,17,18,19,20,21,22,23. However, the origin of these excess capacities are often unclear and attributed to the effects of nano-sized SnS_2_ and possible synergistic effects with carbonaceous materials such as amorphous carbon^11^,^12^, carbon nanotubes^13^,^24^, and reduced graphene oxides^14^,^15^,^16^,^17^,^18^,^19^,^20^,^25^. The synthesis and formation of nanostructures and nanocomposites is a common strategy to overcome large volume changes that SnS_2_ experience during lithiation and delithiation that plagues the stability and cyclability of the active material. The carbonaceous materials within the nanocomposite does not only provide volume change buffers for SnS_2_, but also serves to improve electron conduction. These strategies are also common to transition metal oxides and sulfides based anode materials^26^,^27^,^28^,^29^,^30^. However, efforts have been limited to the above strategies and little has been done to properly utilize both conversion (equation 2) and alloying (equation 4) process of SnS_2,_ which can potentially improve capacities significantly.

Herein, we present the successful integration of a small amount of Molybdenum (Mo) nanoparticles catalyst, directly onto SnS_2_ nanosheets, to promote the active reversible conversion process of Sn to SnS_2_ and decomposition of Li_2_S. Mo catalyst, grown onto SnS_2_ in the form of MoS_2_, leads to a remarkable enhancement in the performance of the lithium ion batteries. Furthermore, SnS_2_ nanosheets are grown onto 3-dimensional graphene foam (3DG) to improve conductivity and suppress volume changes during charge and discharge cycles. The as-synthesized SnS_2_/MoS_2_/3DG are directly used as binder-free and lightweight electrodes where it exhibits enhanced electrochemical performance in terms of improved specific capacity, high rate capability and cycling stability. Direct evidence of the catalytic effect of Mo is also observed via *ex situ* transmission electron microscopy (TEM) and X-ray photonspectroscopy (XPS) analysis. This catalytic process is able to overcome the irreversible reaction in the tin-based anode materials and has important implications for future applications for such materials in various energy storage systems.

## Results and Discussion

### Characterization of Materials

**Figure 1:**
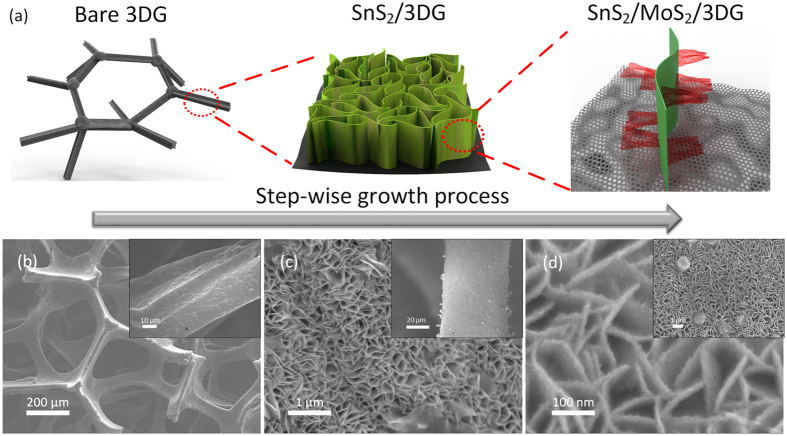
(**a**) Illustration of the growth process of SnS_2_/MoS_2_/3DG via stepwise solvothermal synthesis. In increasing high and low (inset) magnification SEM images of (**b**) pristine etched 3DG, (**c**) SnS_2_/3DG, and (**d**) SnS_2_/MoS_2_/3DG.

**Figure 2:**
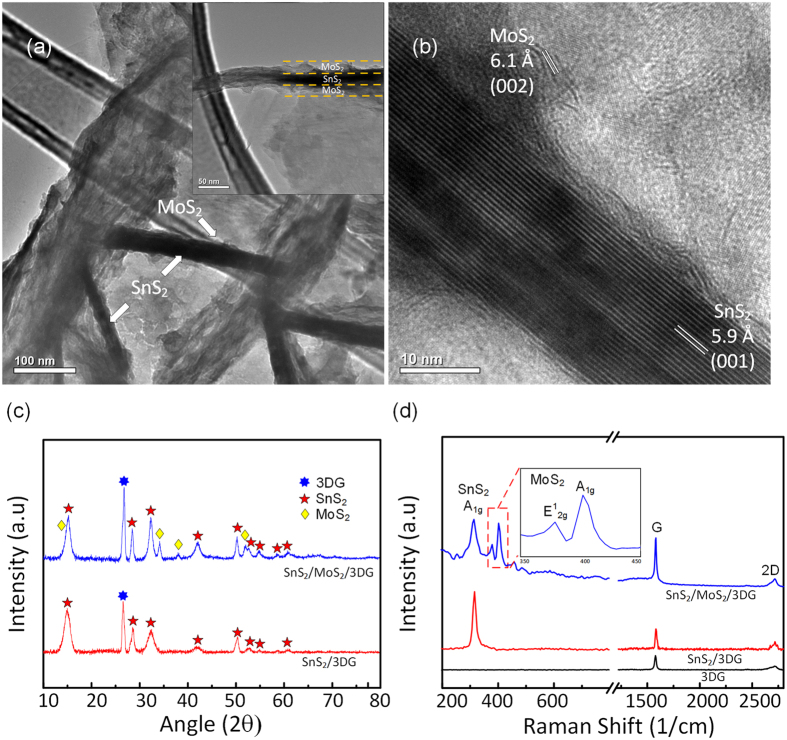
HRTEM images of SnS_2_/MoS_2_/3DG (**a**) low magnification with inset showing sandwich MoS_2_/SnS_2_/MoS_2_ and (**b**) high magnification showing lattices of SnS_2_ and MoS_2_. (**c**) XRD pattern of SnS_2_/MoS_2_/3DG and SnS_2_/3DG. (**d**) Raman spectra of SnS_2_/MoS_2_/3DG, SnS_2_/3DG, and as-prepared 3DG.

The step-by-step synthesis process and morphology of SnS_2_/MoS_2_/3DG nanostructure is detailed in the illustration and the corresponding SEM images in Fig. 1. 3DG was prepared by CVD using ethanol as carbon source and Ni foam as a substrate. The Ni foam substrate was subsequently etched away using 3 M HCl leaving a lightweight 3DG current collector (~0.5 mg cm^−2^) (Fig. 1b). SnS_2_ nanosheets (~1.2 mg cm^−2^) were uniformly grown onto 3DG through a facile solvothermal synthesis process where surfactant (SDS), was used to reduce the size of the nanosheet (Fig. 1c)^31^,^32^. This creates a denser loading of SnS_2_ on 3DG (Figure S1, see Supporting Information). In the second solvothermal synthesis step, trace amounts of MoS_2_ precursors were used to control the limited growth of MoS_2_ catalyst on SnS_2_. This was carried out by placing pieces of the as-synthesized SnS_2_/3DG in the Teflon-lined autoclave with the dissolved MoS_2_ precursors. As shown in Fig. 1d, sparse amount of MoS_2_ nanosheets were grown directly onto the surfaces of SnS_2_ nanosheets. Instead of directly introduction Mo as a catalyst, MoS_2_ nanosheets were grown on SnS_2_ to exploit the 2D nature of MoS_2_ with large exposed surfaces that will facilitate rapid catalytic action during charge-discharge cycles. Lastly, ethanol solvent was used in both solvothermal syntheses as 3DG is hydrophobic. The final product, SnS_2_/MoS_2_/3DG, can be used directly as a lightweight binder-free anode material without needing additional conductive carbon. Figure S2 shows a photograph of the as-synthesized products where the stepwise growth of the SnS_2_ and MoS_2_ results in a color change of the electrode, green (SnS_2_) then black (MoS_2_). In order to obtain a more detailed morphology of MoS_2_ growth on SnS_2_, TEM characterization was carried out as shown in Fig. 2a,b. From the TEM image in Fig. 2a, fine sheets of MoS_2_ can be clearly observed on both sides of the larger SnS_2_ nanosheets. This sandwich structure is clearly marked out as shown in the inset of Fig. 2a. In the high resolution TEM image in Fig. 2b, the interplanar spacing of 6.0 Å and 5.8 Å can be assigned to the (002) and (001) crystal planes of MoS_2_ and SnS_2_ respectively^8^,^28^.

The crystalline phases and structures of the as-synthesized SnS_2_/3DG and the final product SnS_2_/MoS_2_/3DG were confirmed via X-ray diffraction (XRD) as shown in Fig. 2c. The major peaks at 15.0°, 28.5°, and 32.2° are respectively indexed to the (001), (100), and (101) of SnS_2_ (JCPDS No. 23-0677)^8^,^33^, while the intense peak at 26.8° arises from the (002) crystal plane of graphitic carbon (JCPDS No. 75-1621)^29^. On the other hand, the new peaks at 34.3°, 38.0°, and 51.9° of the SnS_2_/MoS_2_/3DG can be assigned MoS_2_ (JCPDS No. 65-3656)^34^. Raman spectroscopy was carried out to further identify the as-synthesized materials (Fig. 2d). The peak at 314 cm^−1^ is attributed to the A_1g_ mode of SnS_2_^35^, while the peaks at 378 and 405 cm^−1^ arises from the in-plane E^1^_2g_ and out-of-plane A_1g_ modes of MoS_2_^26^,^36^. In addition, the peaks at 1573 and 2718 cm^−1^ corresponds characteristic G and 2D bands of the 3DG^37^,^38^. Furthermore, the absence of the D band is indicative of the high quality and low defect of 3DG produced via CVD method^39^. Thermogravimetric analysis (TGA) was carried out, at a temperature range of 25 °C to 900 °C in dry air at a rate of 5 °C min^−1^, to quantify the composition and verify the loading of MoS_2_ on SnS_2_/MoS_2_/3DG nanocomposite. From Figure S3, the initial weight loss of ~5% arises from the moisture loss from the surfaces of the samples. The second step between 200 to 500 °C is attributed to the oxidation of SnS_2_ and MoS_2_^28^,^40^. As SnS_2_ and MoS_2_ oxidize at roughly the same temperature range, mass loading of MoS_2_ in SnS_2_/MoS_2_/3DG can be deduced by the difference in weight loss (~3%) between the two samples (SnS_2_/3DG vs. SnS_2_/MoS_2_/3DG). Finally, the last weight loss step between 600 to 850 °C corresponds to the combustion of 3DG in air^29^. Therefore, based on TGA, the composition of SnS_2_/MoS_2_/3DG and be deduced to be SnS_2_: 0.65, MoS_2_: 0.03, 3DG: 0.32 [See supporting information for detailed derivation].

**Figure 3:**
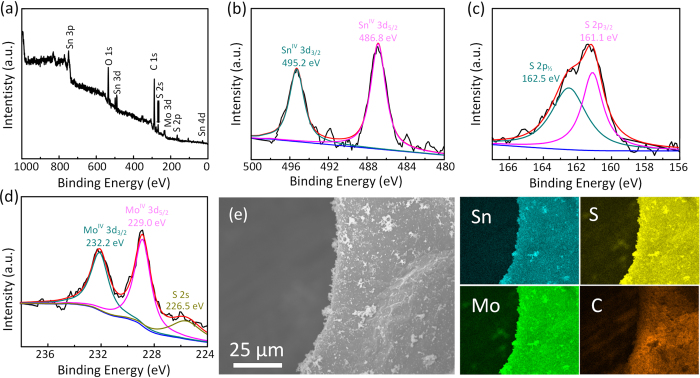
(**a**) XPS survey spectrum, (**b**) Sn 3d spectra, (**c**) S 2p spectra, and (**d**) Mo 3d spectra of SnS_2_/MoS_2_/3DG. (**e**) SEM image of SnS_2_/MoS_2_/3DG corresponding to the EDX elemental mapping images of Sn, S, Mo, and C showing uniform distribution of the elements.

The chemical state and elemental distribution of SnS_2_/MoS_2_/3DG was also confirmed by X-ray photoelectron spectroscopy (XPS) and elemental dispersive X-ray (EDX) analysis, respectively. Figure 3a shows the broad spectrum of the XPS scan on SnS_2_/MoS_2_/3DG where the presence of elements Sn, S, Mo, and C are clearly observed. The high resolution XPS spectra of these elements are shown in Figs 3b,c,d, and S4, respectively. The deconvolution of these high resolution spectra produced pairs of peaks at 495.2 and 486.8 eV (Fig. 3b), 162.5 and 161.1 eV (Fig. 3c), and 232.2 and 229.0 eV (Fig. 3d), corresponding to the Sn 3d_3/2_ and Sn 3d_5/2_ states in SnS_2_, S 2p_1/2_ and S 2p_3/2_ in MoS_2_ and SnS_2_, as well as Mo 3d_3/2_ and Mo 3d_5/2_ in MoS_2_, respectively^22^,^41^. The results indicate the oxidation states of Sn and Mo to be +4, which are in good agreement with the phase pure results of SnS_2_/MoS_2_/3DG obtained via XRD. The deconvuluted C 1 s spectra in Figure S4 shows the presence of several oxygen functional groups on the surfaces of 3DG such as C-O/C-O-C at 285.2 eV as well as C=O/O-C=O at 288.5 eV^42^. In addition, EDX analysis in Fig. 3e shows homogenously distributed elements, Sn, Mo, S, and C, which implies the uniform growth of SnS_2_/MoS_2_ on the surfaces of 3DG.

### Electrochemical Measurements

**Figure 4:**
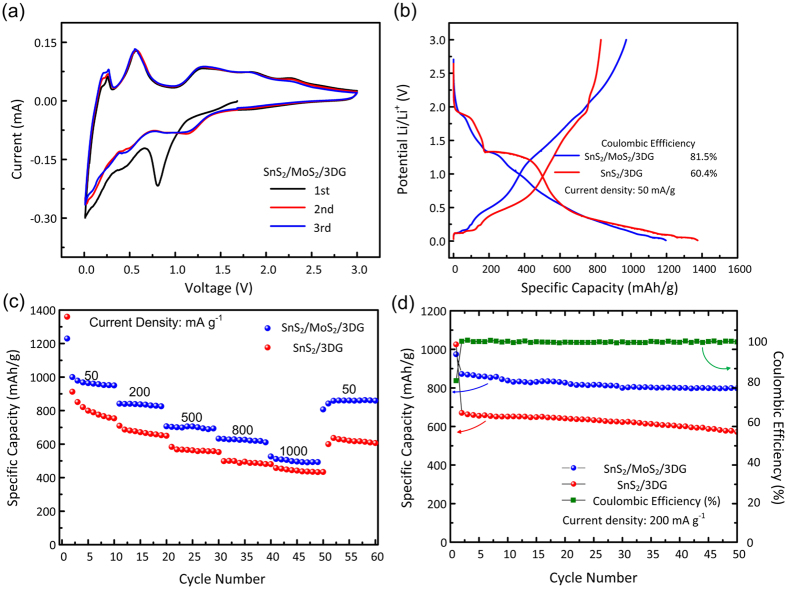
(**a**) Cyclic voltammograms of SnS_2_/MoS_2_/3DG electrode at a rate of 0.05 mV s^−1^ in a potential range of 0.01 to 3.0 V. (**b**) Galvanostatic discharge and charge curves for SnS_2_/MoS_2_/3DG and SnS_2_/3DG electrodes at the 1^st^ cycle at a current density of 50 mA g^−1^ in the potential range of 0.01 to 3.0 V showing the initial Coulombic Efficiency. (**c**) Rate capability of SnS_2_/MoS_2_/3DG and SnS_2_/3DG electrodes. (**d**) Cycling performance of SnS_2_/MoS_2_/3DG and SnS_2_/3DG electrodes at current density of 200 mA g^−1^, and the corresponding coulombic efficiency of SnS_2_/MoS_2_/3DG electrode.

The catalytic effect of Mo in SnS_2_/MoS_2_/3DG was verified via electrochemical characterization through cyclic voltammetry (CV) and galvanostatic charge-discharge cycling. These experiments were carried out through the assembly of a half-cell battery with SnS_2_/MoS_2_/3DG as a binder-free electrode and lithium foil as a counter electrode. Control sample, SnS_2_/3DG, was tested in the same conditions. Figure 4a shows the CV curves of the SnS_2_/MoS_2_/3DG electrodes. In the initial discharge cycle, the cathodic small broad peak centered at around 1.5 V can be attributed to the initial Li insertion into SnS_2_ and MoS_2_, without phase transformation, forming Li_x_SnS_2_ and Li_x_MoS_2_ 9,43. Upon further discharge, peaks at ~0.8 V and ~0.45 V can be ascribed to the conversion reaction of lithiated SnS_2_ and MoS_2_ to metallic Sn and Mo, respectively^16^,^43^. Towards the end of the discharge cycle, the broad peak from ~0.1–0.3 V corresponds to the alloying reaction between Sn and Li followed by the insertion of Li into 3DG, as well as the formation of the solid-electrolyte interface (SEI) film between the active materials and electrolyte. The formation of the SEI film contributes significantly to the initial loss in discharge capacity^9^,^29^,^44^. In the following charge-discharge cycles, the overlapping redox peaks can be attributed to the highly reversible lithium insertion/extraction, conversion and alloying reactions. The first redox pairs at ~0.1/0.25 V and ~1.2/1.3 V arise from the lithium insertion and extraction in 3DG and MoS_2_, respectively^28^,^29^. Next, the alloying and de-alloying reactions of Sn with Li produces redox peaks centered around 0.25 V and 0.56 V, respectively^33^. Lastly, the redox pairs at ~1.16/1.86 V and ~1.8/2.26 V correspond to the conversion reactions occurring for both Sn and Mo, respectively^14^,^28^. In more details, the anodic peaks represent the reduction of SnS_2_ and MoS_2_ to metallic Sn and Mo as well as formation of Li_2_S. The cathodic peaks represent the reformation of SnS_2_ and MoS_2_ with the decomposition of Li_2_S. In the absence of the catalyst, Mo, the CV curves of SnS_2_/3DG are similar to that of SnS_2_/MoS_2_/3DG with the exception of the peaks arising MoS_2_, as well as a gradually decaying redox pair ~1.32/1.87 V (Figure S5). The observed weakened redox pair indicates that the conversion reaction is only partially reversible in SnS_2_/3DG. In contrast, the strong overlapping of the Sn conversion reaction redox pairs for SnS_2_/MoS_2_/3DG implies that in the presence of Mo catalyst, the conversion process of Sn becomes highly reversible^14^. The reactions can be described using the following equations^9^,^28^:

Reactions involving SnS_2_

In the first discharge,


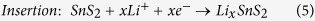



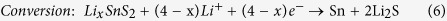


Simplifying equations (5) & (6),


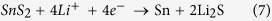


In subsequent charge-discharge cycles,


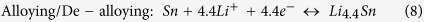


Reactions involving MoS_2_


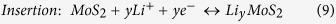



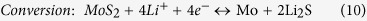


Where *x* and *y* are the number of moles of Li^+^/e^−^ involved in the insertion reactions with SnS_2_ and MoS_2_ respectively.

Figure 4b shows the initial charge-discharge profile of both SnS_2_/MoS_2_/3DG and SnS_2_/3DG. Similar to the CV curves, both electrodes display similar charge-discharge profile with differences owing to the presence of MoS_2_ in SnS_2_/MoS_2_/3DG. The first discharge begins with a brief slope at ~1.8 V followed by a short plateau at ~1.3 V which is attributed to the insertion of Li ion (equations 5 & 9) into and subsequent conversion of SnS_2_ and MoS_2_ (equations 6 & 10), respectively^9^,^43^. Thereafter, the curve proceeds with a gentle slope towards 0.05 V followed by small plateau. The sloped region corresponds to the alloying process of Li with Sn (equation 8) as well as the formation of the SEI layer which results in an irrecoverable loss of lithium^9^,^44^. The small plateau at 0.05 V arises from the intercalation of Li ions to 3DG^29^. In the charge cycle, delithiation proceeds with extraction of Li ions from 3DG at ~0.15 V followed by de-alloying of Li_4.4_Sn at ~0.5 V. Upon charging to higher voltage (1.0–3.0 V), Li_x_MoS_2_ delithiates (equation 9), Sn oxidizes to SnS_2_ while Li_2_S decomposes to S (backward reaction in equation 7)^14^,^43^. However, in the case of SnS_2_/3DG, the oxidation of Sn and decomposition of Li_2_S is limited, which can be observed in the acute slope between 1.9–3.0 V. Therefore, the catalytic effect of MoS_2_ in SnS_2_/MoS_2_/3DG is evident with the reversible decomposition of Li_2_S and oxidation of Sn to SnS_2_ (equation 7). This is in stark contrast to previously reported SnS_2_ electrodes where Li_2_S formed during the initial discharge is irreversible or at most partially reversible in the first few cycles^9^. The reversible decomposition of Li_2_S in the first cycle leads to an improved initial Coulombic Efficiency (ICE). This can be observed between the MoS_2_ loaded, SnS_2_/MoS_2_/3DG with 81.5% CE (first discharge capacity: 1196.5 mA h g^−1^, first charge capacity: 973.0 mA h g^−1^) compared to SnS_2_/3DG with 60.4% CE (first discharge capacity: 1372 mA h g^−1^, first charge capacity: 829.7 mAh g^−1^). The higher first discharge capacity in the SnS_2_/3DG electrode may be due to a larger formation of SEI layer. The effective catalytic conversion reaction of SnS_2_ may be attributed to the direct growth of MoS_2_ on SnS_2_ nanosheets that facilitate rapid reversible conversion process of Sn and decomposition of Li_2_S.

Apart from improving the ICE, the reversible decomposition of Li_2_S increases the total number of moles of Li ion reacting with SnS_2_ from 4.4 (equation 8) to 8.4 (equations 7 and 8) by utilizing both alloying and conversion processes. In this manner, the theoretical capacity of SnS_2_ increases significantly from 644 mA h g^−1^ (4.4 mols of Li^+^) to 1231 mA h g^−1^. In the case of SnS_2_/MoS_2_/3DG, the theoretical capacity of the nanocomposite, taking into the account of MoS_2_ and 3DG, should be 940 mAh g^−1^ (Capacity_SnS2/MoS2/3DG_ = 1231 ∗ 0.65 + 670 ∗ 0.03 + 372 ∗ 0.32). This increase is evident even at different current densities as observed in Fig. 4c where the two different samples are subjected to galvanostatic charge-discharge cycles at increasing current densities. SnS_2_/MoS_2_/3DG delivered discharge capacities of 960.5, 837.0, 705.1, 626.2, and 495.6 mA h g^−1^ at current densities of 50, 200, 500, 800, and 1000 mA g^−1^, respectively. This is distinctly higher as compared to SnS_2_/3DG with highest reversible discharge capacities of ~800 mA h g^−1^ at low current densities of 50 mA g^−1^ and high current discharge capacity of 441.7 mA h g^−1^ at 1000 mA g^−1^. Highly reversible and stable cycling was also observed for the MoS_2_ loaded SnS_2_/MoS_2_/3DG at current density of 200 mA g^−1^ where capacity retention is 91.5% after 50 cycles (Fig. 4d). Furthermore, the first cycle CE is also consistent with that at 50 mA g^−1^ of 82% that increased and maintain near 100% in subsequent cycles (Fig. 4d). The high capacity, highly reversible and stable cycling implies the effective catalytic action of MoS_2_. Furthermore, successful reversible decomposition of Li_2_S at high current densities of 1000 mA g^−1^ highlights importance of the sandwiched MoS_2_/SnS_2_/MoS_2_ nanostructure.

Electrochemical impedance spectroscopy (EIS) measurements were also conducted for both SnS_2_/MoS_2_/3DG and SnS_2_/3DG to understand and evaluate the influence of MoS_2_ loading on SnS_2_/3DG. The measurements were performed on the samples at a semi-charged state (~2.5 V) across a frequency range of 10 mHz to 1 MHz.

**Figure 5:**
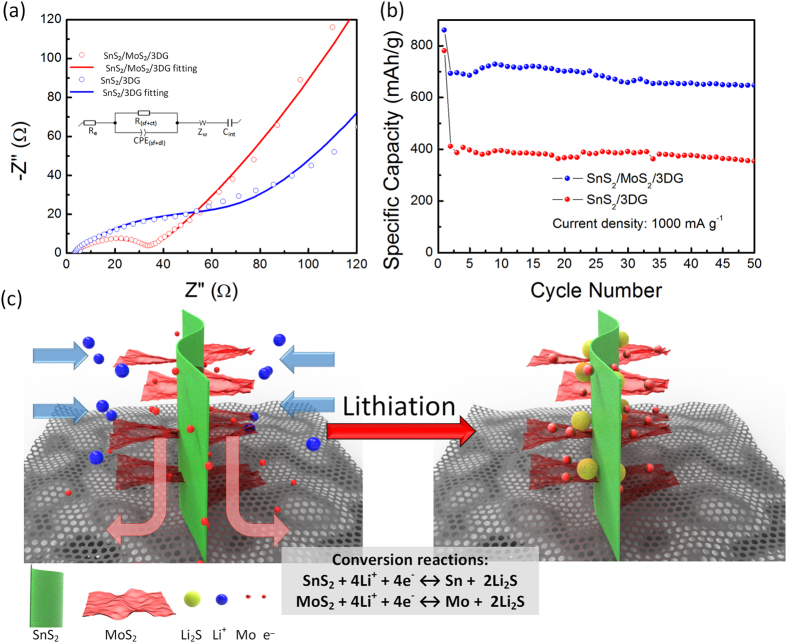
(**a**) Electrochemical impedance spectra of SnS_2_/MoS_2_/3DG and SnS_2_/3DG electrodes as well as the corresponding fitting for both electrodes. Inset: Equivalent circuit used for curve fitting. (**b**) Cycling performance of SnS_2_/MoS_2_/3DG and SnS_2_/3DG electrodes at current density of 1000 mA g^−1^. (**c**) Illustration of MoS_2_/SnS_2_/MoS_2_ sandwich structure which is able to efficiently promote catalytic action.

Figure 5a shows the Nyquist plots where the curves from both electrodes display similar profile; a small semi-circle in the high frequency region corresponding to a combination of surface film resistance (R_sf_) and charge transfer resistance (R_ct_) followed by an acute straight line in the low frequency region which arises from the Warburg region (Z_w_) as well as a Li accumulation element (C_int_)^30^,^45^. An equivalent circuit consisting of R_sf+ct_, Z_w_, C_int_, as well as R_e_, which is the ohmic resistance arising from the electrolyte and cell components, and CPE_(sf+dl)_, a constant phase element relating to the surface film and double layer, was used to fit the Nyquist plots (inset in Fig. 5a). The fitted values using the equivalent circuit are presented in Table S1. The lower surface film and charge transfer resistance (R_sf+ct_) of SnS_2_/MoS_2_/3DG (33.65 Ω) compared to SnS_2_/3DG (64.67 Ω) is an indication that MoS_2_ loading improves charge kinetics. This improvement may be attributed to the release of conductive metallic Mo, through the conversion process of MoS_2_ in the SnS_2_/MoS_2_/3DG electrode, over charge-discharge cycle. The effects of the enhanced conductivity via MoS_2_ loading in SnS_2_/MoS_2_/3DG can be observed by evaluating the electrode at high current densities where charge kinetics are more demanding. Figure 5b shows the discharge capacities of SnS_2_/MoS_2_/3DG and SnS_2_/3DG at high current densities of 1000 mA g^−1^ for 50 cycles where the capacity retention was 85.2%. Owing to the enhanced conductivity and charge kinetics, rapid catalytic action of Mo is achievable. This is reflected by the substantially increased capacity of the SnS_2_/MoS_2_/3DG (647 mA h g^−1^) as compared to SnS_2_/3DG (355.1 mA h g^−1^) (Fig. 5b). Another key contributing factor to the effective catalytic action of Mo at different current densities is the well-designed SnS_2_/MoS_2_/3DG hierarchical nanostructure as illustrated in Fig. 5c. The sandwich MoS_2_/SnS_2_/MoS_2_ nanostructure allows the released metallic Mo to be in direct proximity of Li_2_S formed during lithiation. In this manner, Mo is able to rapidly catalyze the decomposition of Li_2_S during delithiation.

### Further evidence towards catalytic effect of Mo

In order to further demonstrate the catalytic effect of Mo, control samples of SnS_2_ and SnS_2_/MoS_2_ were synthesized in the same method as SnS_2_/3DG and SnS_2_/MoS_2_/3DG in the absence of 3DG, respectively. Without the 3DG current collectors to act as a platform for ordered growth, the as-synthesized SnS_2_ nanosheets aggregated into micron sized clusters, which resembled a fully bloomed flower (Figure S6a). When MoS_2_ was introduced, the nanosheets of MoS_2_ was also able to grow on the surfaces of SnS_2_ flowers (Figure S6b). Given the lack of a growth platform, the as-synthesized control samples were in the form of powders. Hence, to obtain an electrode, these powders were made into a slurry form that were subsequently coated on Ni-foam current collectors and finally assembled into coin cells. Similarly, CV and galvanostatic cycling were performed on SnS_2_ and SnS_2_/MoS_2_ as shown in Figure S6c–f. The CV peaks of SnS_2_ and SnS_2_/MoS_2_ are consistent with SnS_2_/3DG and SnS_2_/MoS_2_/3DG, respectively, with the exception of peaks arising from 3DG. In terms of galvanostatic cycling, the catalytic effect could also be observed in SnS_2_/MoS_2_ where there is a significant improved of ICE from 55% (SnS_2_) to 67% (SnS_2_/MoS_2_) (Figure S6e) as well as improved discharge capacity from 6745 mAh g^−1^ (SnS_2_) to 806 mAh g^−1^ (SnS_2_/MoS_2_) (Figure S6f). However, at higher current densities (500, 800, and 1000 mA g^−1^), both electrodes delivered roughly the same discharge capacities. This could be due to the instability of the SnS_2_/MoS_2_ structure that resulted in the gradual dissociation of MoS_2_ nanosheets from SnS_2_ surfaces because of significant volume changes of SnS_2_ after prolonged cycling. Therefore, these results further supports the presence of the catalytic action of Mo towards the decomposition of Li_2_S and oxidation of Sn to SnS_2_ which results in a substantial improvement towards both the ICE and capacities of SnS_2_ anodes. Furthermore, despite the improvement, the rapid decay in capacities of SnS_2_/MoS_2_ highlights the importance of a rational design towards a well-structured composite that is demonstrated in SnS_2_/MoS_2_/3DG.

Thus far, there has already been numerous reports citing high discharge capacities in SnS_2_-based anode beyond its 644 mAh g^−1^ theoretical capacity^14^,^15^,^16^,^17^,^18^,^19^,^20^,^21^. In most of these cases, SnS_2_ was synthesized as a nanocomposite together with reduced graphene oxide (rGO). For example, Lin *et al*. reported the synthesis of ultrasmall SnS_2_ on rGO with high capacities of 1034 mA h g^−1^ at current density of 64.5 mA g^−1^ while few-layer SnS_2_/graphene presented by Chang *et al*. delivered capacities of 920 mA h g^−1^ 16,46. However, the contributing factors towards the excess capacity are loosely assigned to a synergistic effect between SnS_2_ and rGO without clear clarification. In a recent report, Qu *et al*. attributed the high capacities of the SnS_2_/Graphene nanocomposite to a reversible conversion of SnS_2_ from Sn through a catalytic effect brought about by Sn nanoparticles, which were released during conversion reactions of SnS_2_, stacked within graphene matrices^14^. Unfortunately, there was no physical evidence provided to support this postulation. Furthermore, due to the presence of defects, surface functional groups and other additional Li storage sites, the capacity of rGO varies depending on the quality of the graphene sheets and can be as high as ~1000 mA h g^−1^ 47. As a result, the capacity contribution by rGO in the SnS_2_/rGO nanocomposites may be more significant than reported.

In an analogous system, the reversible decomposition and formation of Li_2_O from SnO_2_ and GeO_2_ has been shown to be mediated in the presence of nano-sized catalyst, Co and Ge respectively. This led to capacities beyond their theoretical capacities (SnO_2_/Co_3_O_4_/rGO: 1038 mAh g^−1^ vs SnO_2_: 782 mAh g^−1^ and GeO_2_/Ge/C: 1860 mAh g^−1^ vs GeO_2_: 1126 mAh g^−1^)^48^,^49^,^50^,^51^,^52^. The enhanced capacities were attributed to the catalytic role of the respective dopants which facilitated the reversible formation and decomposition of Li_2_O. Another consequence of this catalytic effect is the improvement of initial Coulombic efficiencies (ICE) as a significant portion of the initial capacity is typically lost due to the formation of irreversible Li_2_O apart from the formation of surface electrolyte interface (SEI). Therefore, since SnS_2_ is analogous to SnO_2_, the effect of the catalyst can be extended to SnS_2_ based anodes.

**Figure 6:**
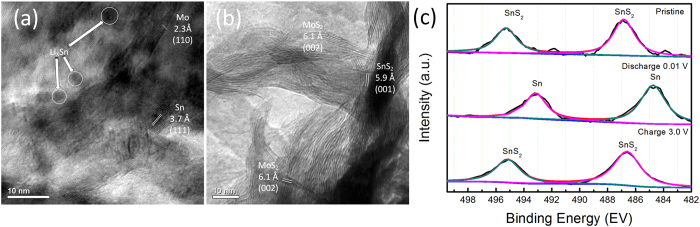
*Ex situ* analysis of post cycled SnS_2_/MoS_2_/3DG electrode. HRTEM image of (**a**) discharge (0.01 V) state and (**b**) charge (3.0 V) state. (**c**) Sn 3d spectra of the electrode in pristine, discharge, and charge state.

The reversible oxidation/reduction of SnS_2_/Sn and Li/Li_2_S as described in equation 7 occurs during the discharge/charge cycles, respectively. Hence, evidence of the reversibility of equation 7 can be investigated through the detection of the products at the end of discharge (0.01 V) and charge (3.0 V) process. In this regard, post cycling TEM and XPS was conducted for SnS_2_/MoS_2_/3DG electrodes after 20 cycles at current density of 200 mA g^−1^. Being free from additives such as binder and active carbon, the cycled electrodes were free from stray and unwanted signals in the post cycling characterizations. Two sets of coin cells were cycled with one set ending at full discharge state (0.01 V) and the other at full charge state (3.0 V). The electrodes were extracted from the coin cell in the Ar-filled glove box, soaked overnight with acetonitrile and washed with ethanol several times. The *ex-situ* HRTEM of the fully discharged and charged electrodes are shown in Fig. 6a,b, respectively. Upon full discharge (lithiation), crystalline lattices corresponding to metallic Mo and Sn can be found (Fig. 6a). This corresponds to the conversion process of MoS_2_ and SnS_2_ to their metallic phases as well as the incomplete lithium alloying of Sn^51^,^53^,^54^. On the other hand, lithiated Sn alloy (Li_X_Sn) which are often reported as nanoparticles embedded within the Sn-based electrodes, may be identified as the dark nanoparticles (~3–5 nm) circled out in Figs 6a and S7^55^,^56^,^57^. However, crystal lattice of the Li_x_Sn nanoparticles could not be clearly observed as they are embedded within the overlapping lattices from Sn and Mo in the complicated SnS_2_/MoS_2_/3DG structure. Li_2_S was not identified due to its amorphous nature at full lithiated state^58^. In the fully charged state (delithiation), the presence of crystalline lattices which can be assigned to MoS_2_ and SnS_2_, implies a reversible conversion process of metallic Mo and Sn as well as Li_x_Sn to their intial state (Fig. 6b). Therefore, Sn formed in the discharge state and SnS_2_ charged state corresponds well with reversibility of equation 7 where the forward reaction corresponds to discharge (formation of Sn) and backward reaction the charge process (formation of SnS_2_). As an additional evidence towards the reversible conversion of SnS_2_, *ex-situ* XPS was also conducted on the cycled electrodes at full discharge and charge states. Figure 6c compares the high-resolution XPS spectra of SnS_2_/MoS_2_/3DG electrodes at different states, pristine, discharged to 0.01 V, and charged to 3.0 V. The pristine electrode corresponds to the initial state of SnS_2_/MoS_2_/3DG where the XPS spectra peaks corresponds to Sn 3d_5/2_ and Sn 3d_3/2_ as discussed in Fig. 3b. Upon discharge, these peaks shift downwards to 493.1 eV and 484.7 eV corresponding the reduction of Sn^4+^ of SnS_2_ to metallic tin (Sn^0^)^59^. In the charge state, the shifted peaks returns to the initial positions of 495.1 eV and 486.6 eV indicating an oxidation of Sn^0^ to Sn^4+^, i.e. the reformation of SnS_2_ 60. Once again, the presence of Sn at discharge states and SnS_2_ at charged states points towards the reversibility of the reaction in equation 7. Therefore, the results obtained from the *ex-situ* HRTEM and XPS analyses of the post cycled electrodes at charged and discharge states provides conclusive evidence that the reversible conversion reactions of SnS_2_ (equation 7) are promoted through the catalytic action of transition metal, Mo, in the active material matrix.

## Conclusion

In summary, a rationally designed hierarchical nanostructured SnS_2_/MoS_2_/3DG nanocomposite was synthesized via stepwise solovothermal synthesis of metal sulfides on a lightweight current collector substrate, 3DG. The stepwise synthesis facilitated the growth of self-assembled vertical SnS_2_ nanosheets on surfaces of 3DG followed by growth of ultrafine MoS_2_ nanosheets which branches out on both sides of SnS_2_, creating a sandwich MoS_2_/SnS_2_/MoS_2_ structure. The sandwich structure places MoS_2_ in very close proximity to SnS_2_ that facilitates rapid catalytic action of Mo towards decomposition of Li_2_S and conversion of SnS_2_. As a catalyst towards the reversible conversion reaction of SnS_2_, the loading of MoS_2_ on SnS_2_/MoS_2_/3DG was controlled at a low content of 3%. A small amount of Mo proved sufficient towards the catalytic effect reversible conversion of SnS_2_ that saw a significantly improved ICE as well as increased discharge capacities even up to high current density of 1000 mA g^−1^. The binder-free SnS_2_/MoS_2_/3DG lightweight electrode also facilitated post cycling investigations. *Ex situ* HRTEM and XPS analysis of charged and discharged electrodes provided substantial evidence towards the catalytic effect of Mo in the reversible conversion of SnS_2_; metallic Sn and Mo could be found in discharged electrodes while SnS_2_ and MoS_2_ identified in charged electrodes. Therefore, the use of catalyst to unlock the potential of SnS_2_ as an anode that exploits both conversion and alloying reactions creates a new direction towards high capacity metal sulfide anode materials.

## Experimental Section

SnS_2_/MoS_2_/3DG was prepared by 3 general steps 1, chemical vapor deposition (CVD) to obtain 3DG 2, solvothermal to obtain SnS_2_ on 3DG (SnS_2_/3DG), and 3 solvothermal to obtain MoS_2_ on SnS_2_/3DG (SnS_2_/MoS_2_/3DG).

### Preparation of 3DG and removal of Ni Foam

The preparation of the 3DG foam adopts a similar method as previously reported^29^,^33^,^61^. Ni foam (1.6 mm thick, purchased from Alantum Advanced Technology Materials (Shenyang)) was cut into 150 mm × 45 mm pieces. The rectangular Ni foam pieces are then rolled and placed into a 1 inch quartz tube. Before heat was applied, Argon (Ar) gas was allowed to flow for 10 mins to remove residual air in the tube. Thereafter, a heat rate of 50 °C min^−1^ was applied to heat the quartz tube to 1000 °C. At this temperature, ethanol vapour was mixed with the flowing N_2_/H_2_ gas through the bubbling of anhydrous ethanol. The mix N_2_/H_2_/ethanol vapour was allowed to flow for 5 mins. Lastly, the quartz tube was allowed to cool to room temperature rapidly (~100 °C/min). The Ni foam on 3DG-Ni was removed in an etchant solution of 3 M HCl at 80 °C. Pure 3DG was washed using deionized (DI) water and ethanol several times before drying at 60 °C. The typical loading of this process yields ~0.5 mg cm^−2^ of 3DG on Ni foam (3DG-Ni).

### Synthesis of SnS_2_/3DG

The synthesis of SnS_2_/3DG follows the previously reported method with the addition of SDS^33^. SnS_2_ grown on 3DG (SnS_2_/3DG) was prepared via a simple solvothermal reaction. SnCl_4_.5H_2_O (32 mM), thioacetamide (TAA) (80 mM), and Sodium dodecyl sulfate (SDS) (0.07 mM) was weighed and dissolved into 35 mL ethanol. Magnetic stirring and mild heat (50 °C) was applied to ensure homogeneity of the precursors. 6 pieces of 3DG (12 mm diameter) was cut and together with the mixture, transferred into a 50 mL Teflon-line stainless steel autoclave. The reaction proceeded by heating the autoclave at 180 °C for 12 h. After the reaction, as-synthesized SnS_2_/3DG was collected by rinsing with DI H_2_O and ethanol, and drying at 60  °C. The loading of SnS_2_ on 3DG was ~1.2 mg. Control sample, pristine SnS_2_, was synthesized by the same method in the absence of 3DG.

### Synthesis of SnS_2_/MoS_2_/3DG

SnS_2_/3DG synthesized in the previous step was used directly in this step after drying at 60 °C. MoS2 grown on SnS_2_/3DG, termed as SnS_2_/MoS_2_/3DG, was prepared by an L-cysteine assisted solvothermal reaction. L-cysteine (0.25 mM) and Na_2_MoO_4_·2 H_2_O (0.03 mM) was weighed and dissolved in 15 mL DI H_2_O. 15 mL of ethanol was added to the continuously stirred mixture. 6 pieces of SnS_2_/3DG was added to the mixture and transferred to a 50 mL Teflon-line stainless steel autoclave. The stainless steel autoclave was heated at 180 °C for 12 h. The as-synthesized SnS_2_/MoS_2_/3DG was collected by rinsing with DI H_2_O and ethanol, and drying at 60 °C. The loading of MoS_2_ on SnS_2_/3DG was between 0.05–0.1 mg. Lastly, the SnS_2_/MoS_2_/3DG was annealed in Nitrogen (N_2_) environment at 400 °C for 2 hours at a slow heating rate of 3 °C min^−1^. Control sample, SnS_2_/MoS_2_, was synthesized following the above method using pristine SnS_2_ in place of SnS_2_/3DG.

### Materials Characterization

The nanostructures and morphology of the as-synthesized samples were imaged under a field-emission scanning electron microscope (FESEM, JSM-7600) and transmission electron microscope (TEM, JEM-2100F). Crystalline phase and structure of the samples were identified by X-ray diffraction (XRD, Bruker D8). Raman spectroscopy was conducted by a confocal Raman setup with a 532 nm lazer excitation (WITec Instruments Corp, Germany). The nanocomposite content breakdown was measured by thermogravimetric analysis (TGA, Shimadzu, DTG-60). The chemical valence states of the samples were investigated with a X-ray photoelectron spectroscope (XPS, PHI Quantera II, Physical Electronics, Adivision of ULCAV-PHI).

### Coin cell assembly and Electrochemical measurements

The as-synthesized SnS_2_/MoS_2_/3DG was used directly as an anode in the two electrode half-cell configuration where lithium metal acts as the counter electrode (cathode). Control samples of SnS_2_/3DG, pristine SnS_2_, and SnS_2_/MoS_2_ were also assembled. Pristine SnS_2_ was synthesized using the procedure described in step 2, in the absence of 3DG whereas SnS_2_/MoS_2_ was synthesized using the procedure described in step 3 by using pristine SnS_2_ instead of SnS_2_/3DG. Electrodes for the powdered control samples, SnS_2_ and SnS_2_/MoS_2_ were made into a slurry and subsequently coated on Ni foam current collectors (12 mm diameter each). The slurry was prepared by mixing 8 parts of active materials to 1 part of polyvinylidene fluoride (PVDF) binder and 1 part of carbon black in the presence of N-Methylpyrrolidone (NMP) solvent. The SnS_2_/3DG was used directly as an electrode. All working electrodes were dried at 120 °C for 12 h before assembly. The coin cell were assembled in an Ar-filled glove box using standard CR 2032 as casing. Each cell consisted of a Celgard 2400 membrane sandwiched between a working electrode (active material) and counter electrode (lithium metal). Electrolyte used was a mixture of 1 M LiPF6 solution in Ethylene Carbonate (EC)/Di-Methyl Carbonate (DMC), 1:1 v/v. Electrochemical measurements, cyclic voltammetry (CV) and electrochemical impedance spectroscopy (EIS) were performed using an electrochemical workstation (VMP3, Biologic, France). While galvanostatic charge-discharge cycles were carried out using a battery analyser (Neware, China).

## Additional Information

**How to cite this article:** Huang, Z. X. *et al*. Unlocking the potential of SnS_2_: Transition metal catalyzed utilization of reversible conversion and alloying reactions. *Sci. Rep.* **7**, 41015; doi: 10.1038/srep41015 (2017).

**Publisher's note:** Springer Nature remains neutral with regard to jurisdictional claims in published maps and institutional affiliations.

## Supplementary Material

Supplementary Information
