# Supplementary material for: Unlocking the potential of SnS2: Transition metal catalyzed utilization of reversible conversion and alloying reactions
Source: Sci Rep. 2017 Jan 19;7:41015. doi: 10.1038/srep41015 (PMC5244482; doi:10.1038/srep41015)
Supplement: Supplementary Information [file srep41015-s1.doc]

Supporting Information

**Unlocking the potential of SnS2: Transition metal catalyzed utilization of reversible conversion and alloying reactions**

Zhi Xiang Huang,1,2 Ye Wang,1 Bo Liu,1 Dezhi Kong,1 Jun Zhang,3 Tupei Chen,3 Hui Ying Yang[[1]](#footnote-2)

1. Pillar of Engineering Product Development, Singapore University of Technology and Design, 8 Somapah Road, Singapore 487372, Singapore
2. Airbus Group Innovations Singapore, 110 Seletar Aerospace View, Singapore 797562
3. School of Electrical and Electronic Engineering, Nanyang Technological University, Singapore 639798, Singapore


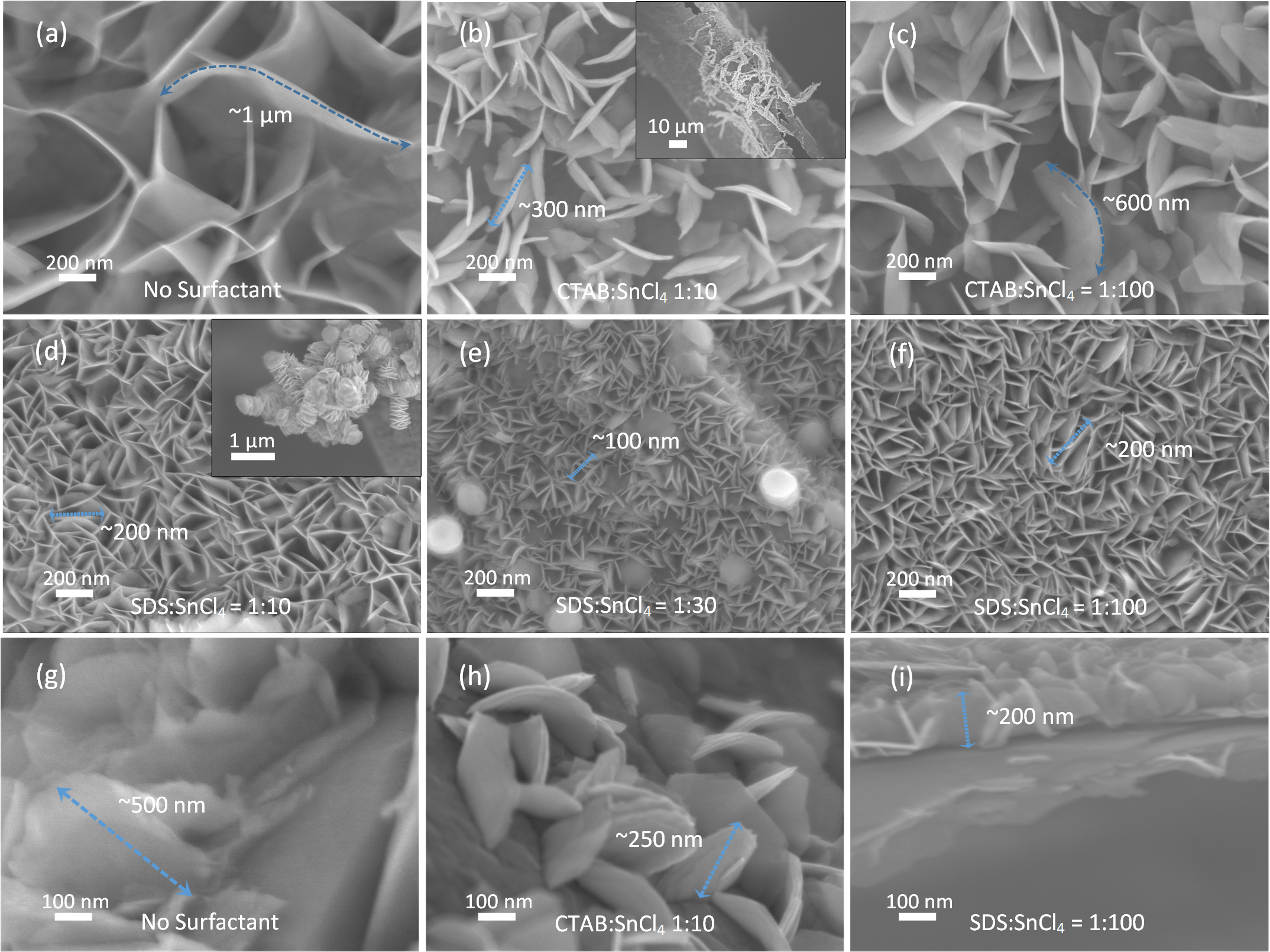


**Figure S1** SEM images of SnS2/3DG synthesized with and without surfactants (SDS or CTAB). (a) no surfactant, CTAB:SnCl4 in ratios of (b) 1:10, (c) 1:100, and SDS:SnCl4 in ratios of (d) 1:10, (e) 1:30, (f) 1:100. SEM images of the different samples indicating the height of the SnS2 nanosheets, (g) no surfactant, (h) CTAB:SnCl4 = 1:10, (i) SDS:SnCl4 = 1:100.

**Effects of Surfactant on SnS2 growth on 3DG**

In a previous work, we reported the synthesis of SnS2/3DG which was prepared via solvothermal of SnCl4 and TAA on 3DG (Figure S1a). In an effort to improve the mass loading of SnS2 on 3DG prior to growth of catalyst (MoS2), surfactants which are known to affect the size and morphology of the synthesized product, were used to refine SnS2/3DG. Two surfactants, namely cationic Cetyltrimethylammonium bromide (CTAB) and anionic sodium dodecyl sulfate (SDS), were attempted. The effects of the surfactants were observed by varying the molar ratio of surfactant to tin precursor (SnCl4). Figure S1b and c shows the effects of CTAB:SnCl4 in molar ratios of 1:10 and 1:100. At high concentrations of CTAB, there was a significant size reduction of SnS2 nanosheet (~300 nm) (Figure S1b). However, during the synthesis of SnS2 on 3DG, a competing reaction was occurring which can be observed by the severe agglomeration of SnS2 (inset of Figure S1b). At lower concentrations of CTAB, size reduction of SnS2 ­­nanosheet was less significant (~600-800 nm) (Figure S1b). On the other hand, at high concentrations of SDS, greater size reduction of SnS2 nanosheet (~200-250 nm) was observed (Figure S1d). Furthermore, an increased density of SnS2 nanosheet can also be clearly seen. However, similar to the case of CTAB, severe agglomeration of SnS2 was also observed (inset of Figure S1d). At lower concentrations of SDS, agglomeration of SnS2 was still present albeit less severe (Figure S1e). The reduced agglomeration was accompanied by a further size reduction of SnS2 nanosheet (~100-150 nm). This could be attributed to the improved utilization of surfactants towards size reduction of SnS2 and growth on 3DG. At low concentrations of SDS, SnS2 nanosheets were about ~200nm. In terms of nanosheet height, the surfactants capped the growth to 250 nm and 200 nm when CTAB and SDS was added, respectively. The width of the nanosheets remained fairly constant at 10 – 20 nm. Amongst the different surfactants and concentrations, SDS at low concentrations (SDS:SnCl4, 1:100) was selected as only a small amount was required to successfully reduce the size of SnS2 nanosheets. Furthermore, the improved packing density of SnS2 left sufficient gap between each nanosheet to facilitate the subsequent growth of MoS2. Owing to the increased density of SnS2 on 3DG, active mass loading of SnS2 was increase by ~20%.


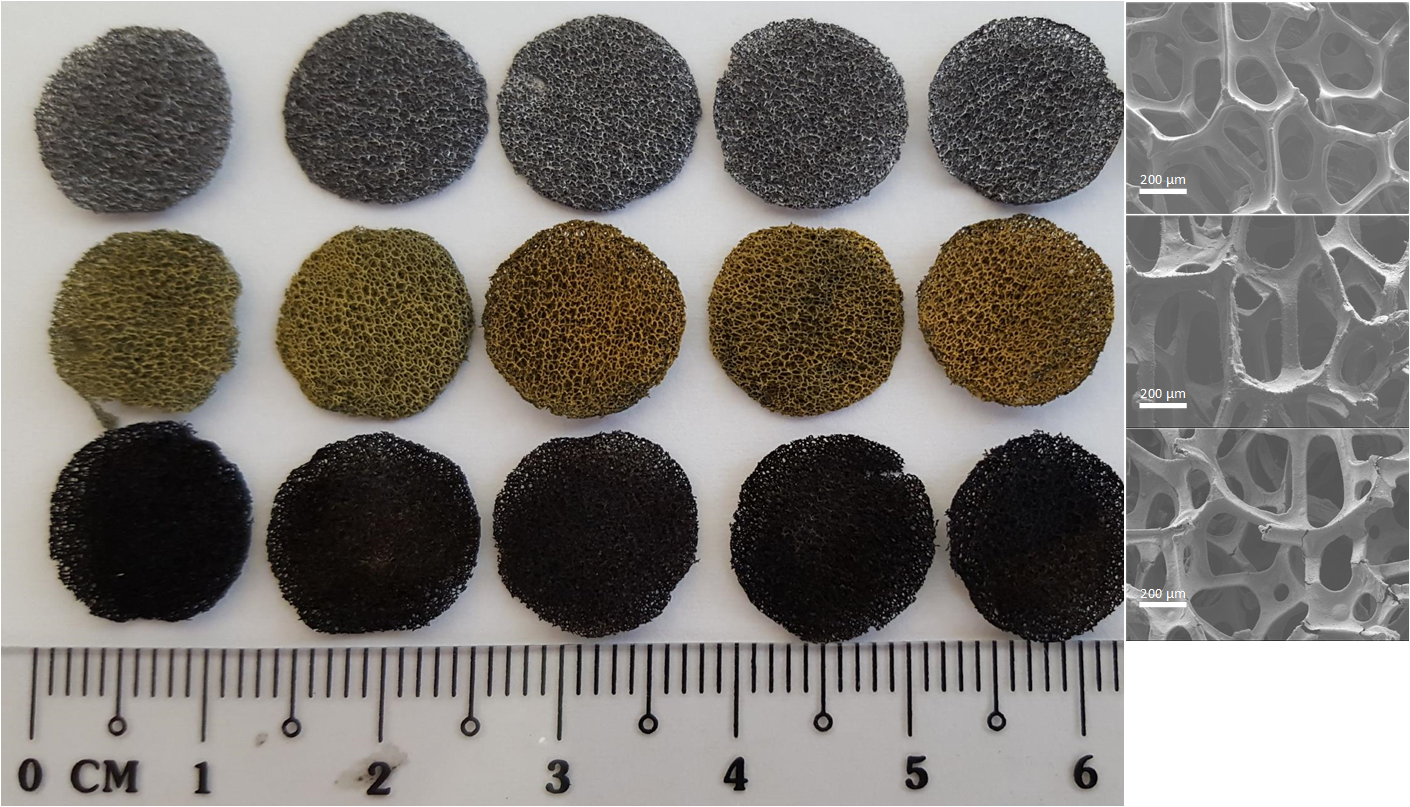


3DG

SnS2/3DG

SnS2/MoS2/3DG

**Figure S2** Photograph and low magnification SEM image of as-prepared materials, top: pristine 3DG after Ni-etch, middle: SnS2/3DG, bottom: SnS2/MoS2/3DG. The etched 3DG are precut into regular 1.2mm diameter discs to facilitate the direct use of the final products in coin-cell as electrodes.


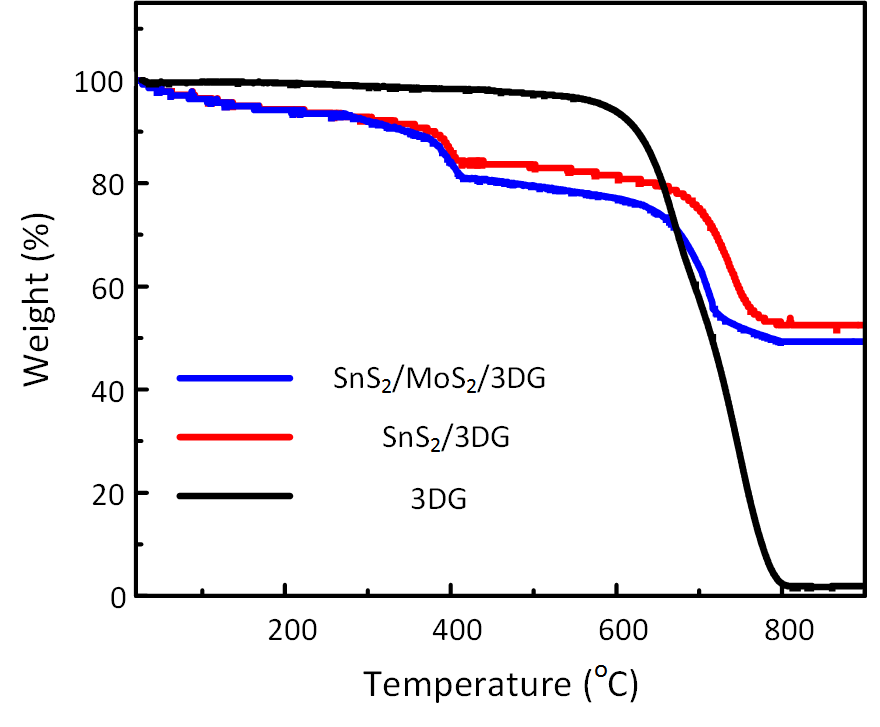


**Figure S3** TGA curves of SnS2/MoS2/3DG, SnS2/3DG, and as-prepared 3DG.

**Calculation of SnS2/MoS2/3DG composition**

As described in the main text and shown in Figure 3C, TGA is used to derive the composition of the as-synthesized SnS2/MoS2/3DG. In order to obtain accurate mass loading of MoS2 and SnS2 on 3DG, a batch of 3DG with identical mass was used to synthesize SnS2/3DG. This process was repeated for the growth of MoS2 on SnS2/3DG. Thereafter, the as-synthesized SnS2/MoS2/3DG and SnS2/3DG was crushed into powder form and used directly for TGA analysis. In this manner, the weight difference between SnS2/MoS2/3DG and SnS2/3DG arises solely from MoS2. As SnS2 and MoS2 oxidizes in air at the same temperature range 200 to 500 oC, it is reasonable to assume that any additional weight loss is due to the oxidation of MoS2. After accounting for the loss of weight due to moisture (~5 %), the weight loss due to oxidation of SnS2 in SnS2/3DG and SnS2 and MoS2 in SnS2/MoS2/3DG was ~12 % and ~15 % respectively. This indicates that the weight loss due to MoS2 is ~3 %. In both samples, weight loss due to the combustion of 3DG is ~ 33 %. Therefore, the mass composition of SnS2/3DG is SnS2 : 3DG = 0.67 : 0.33 while that of SnS2/MoS2/3DG is SnS2 : MoS2 : 3DG = 0.65 : 0.03 : 0. 32.


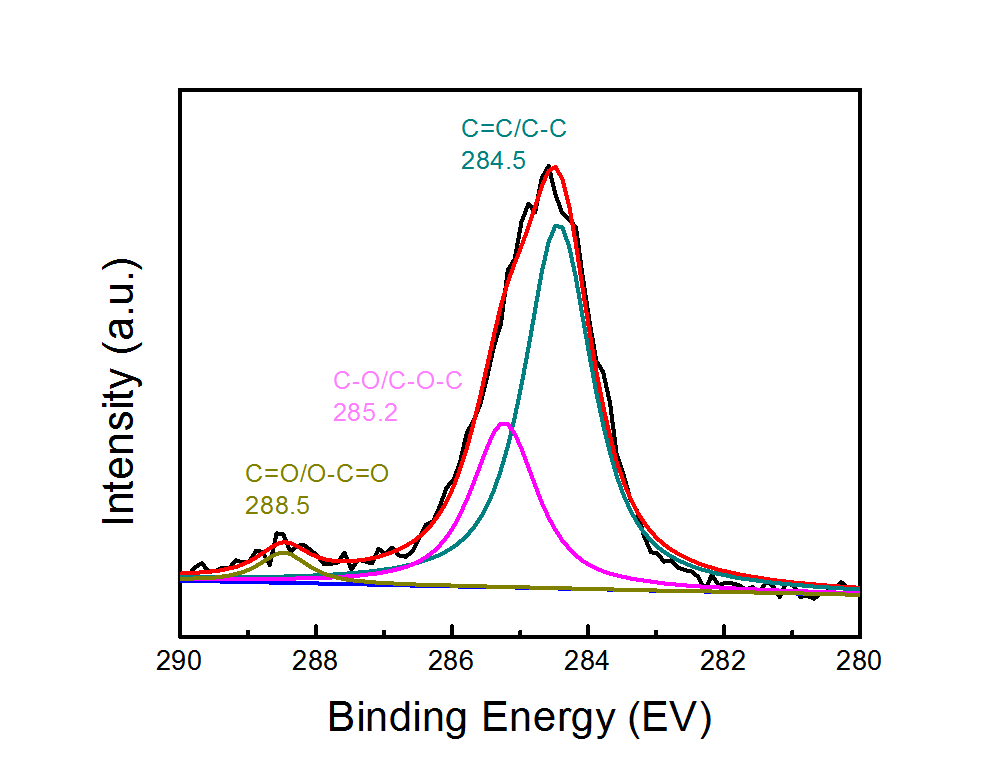


**Figure S4** High resolution XPS spectra of C 1s


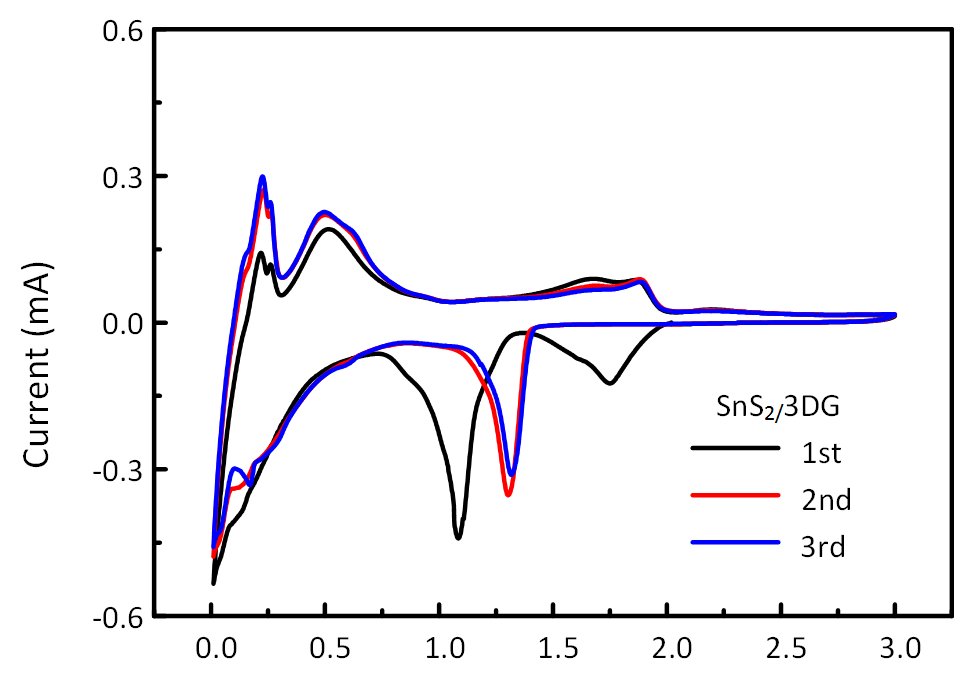


**Figure S5** CV curve of SnS2/3DG electrode in the first 3 cycles at a scan rate of 0.1 mV s-1in a potential range of 0.01 – 3.0 V vs. Li/Li+.


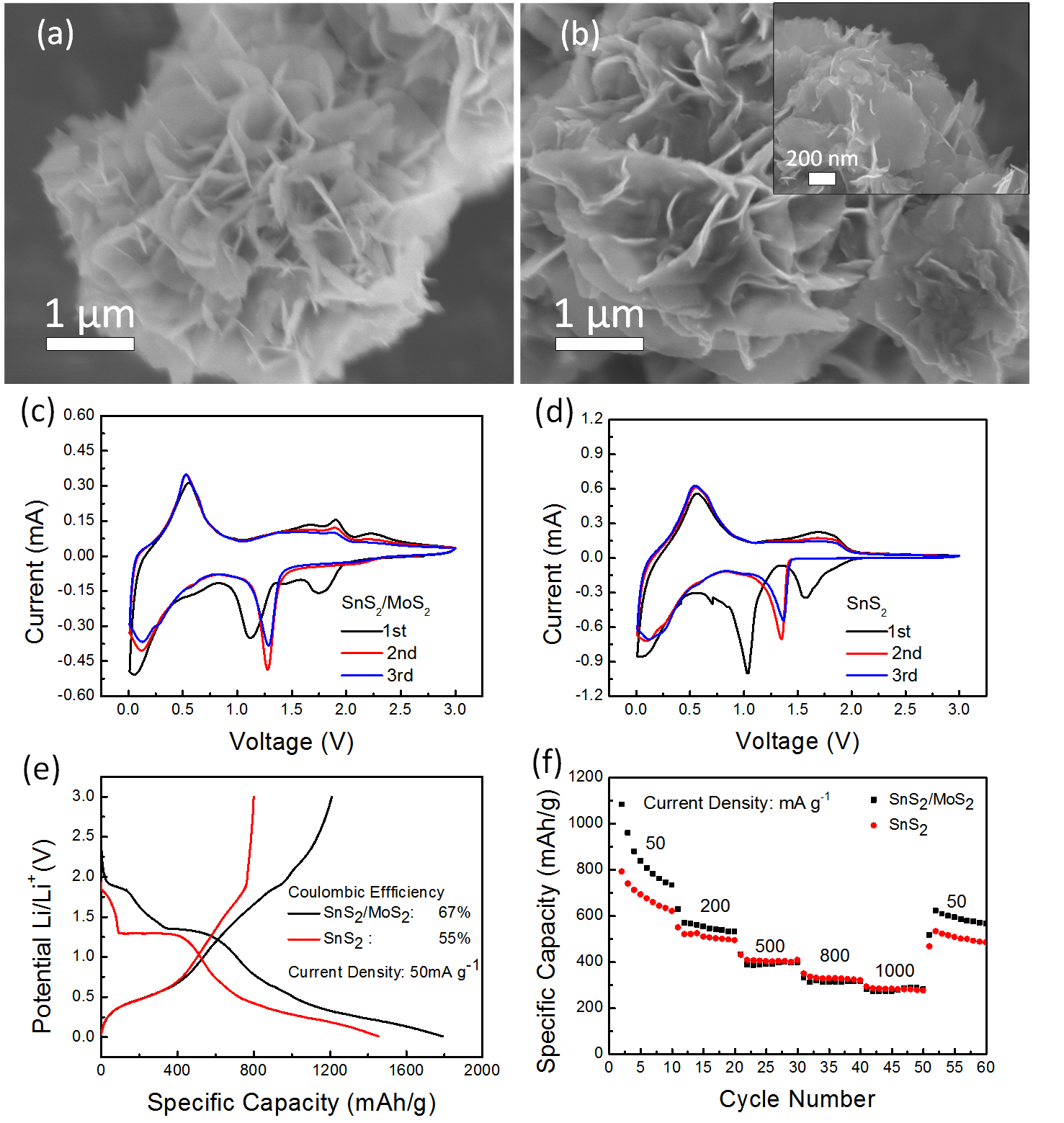


**Figure S6** SEM images of as-synthesized control samples (a) SnS2 and (b) SnS2/MoS2 with inset showing high magnification. Electrochemical performance of control samples SnS2 and SnS2/MoS2. CV curve of (c) SnS2/MoS2 and (d) SnS2 electrode in the first 3 cycles at a scan rate of 0.1 mV s-1in a potential range of 0.01 – 3.0 V vs. Li/Li+. (e) Galvanostatic discharge/charge curves of SnS2/MoS2 and SnS2 electrode for the first cycle at current density of 50 mA g-1. (f) Rate capabilities of the SnS2/MoS2 and SnS2 electrode.

**Table S1**

Fitting results of the EIS curves in Fig 6 using the equivalent circuit

| **Sample** | **Re(Ω)** | **R(sf+ct)(Ω)** |
| --- | --- | --- |
| SnS2/MoS2/3DG | 1.29 | 33.65 |
| SnS2/3DG | 2.85 | 64.67 |
|  |  |  |


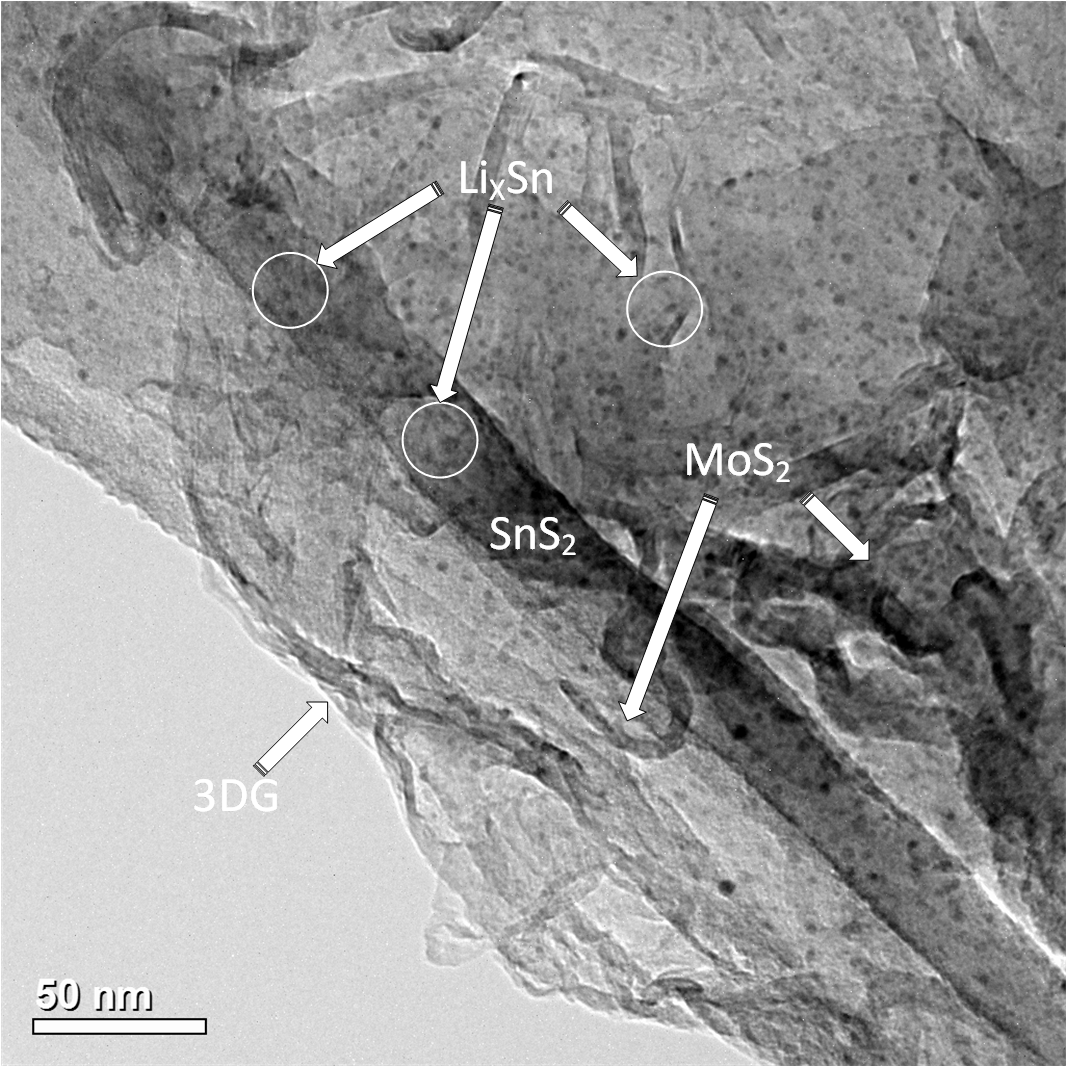


**Figure S7** HRTEM image of post cycled SnS2/MoS2/3DG electrode in full discharge (0.01 V) state.

1. Corresponding author. Tel.: +65 6303 6663; Fax: +65 6779 5161.

   *E-mail address:* [yanghuiying@sutd.edu.sg](mailto:yanghuiying@sutd.edu.sg) (H. Y. Yang) [↑](#footnote-ref-2)
